# Understanding Delivery and Engagement With a Digital Self-Management Intervention for Chronic Obstructive Pulmonary Disease Across Two Clinical Settings: Qualitative Study

**DOI:** 10.2196/88108

**Published:** 2026-09-21

**Authors:** Martin Ruddock, Alison Blythin, Bethany Cliffe, Lucy Yardley, James Dodd, Rachel Williams, Katherine Bradbury, Tom Wilkinson, Ben Ainsworth

**Affiliations:** 1 Faculty of Environmental and Life Sciences, School of Psychology, University of Southampton, University Road, Southampton, England, SO17 1BJ, United Kingdom, 44 23 8059 5000; 2 NIHR Southampton Biomedical Research Centre, Tremona Road, Southampton General Hospital, Southampton, England, United Kingdom; 3 my mhealth Limited, London, United Kingdom; 4 University of Bristol, Bristol, England, United Kingdom; 5 School of Psychological Science, University of Bristol, Bristol, England, United Kingdom; 6 NIHR Applied Research Collaboration West, Bristol, England, United Kingdom; 7 Faculty of Medicine, Academic Respiratory Unit, University of Bristol, Bristol, England, United Kingdom; 8 Liskeard Community Hospital, Respiratory Team, Cornwall Partnership NHS Foundation Trust, Liskeard, United Kingdom; 9 Faculty of Medicine, Clinical and Experimental Sciences, University of Southampton, Southampton, England, United Kingdom

**Keywords:** digital health interventions, engagement, delivery, digital health, real world, evaluation, qualitative

## Abstract

**Background:**

Adherence to chronic obstructive pulmonary disease (COPD) self-management plans can improve health outcomes, yet access to pulmonary rehabilitation remains limited. Digital self-management interventions, such as *myCOPD*, offer a potential means to extend support across care pathways. However, little is known about how these tools are delivered in routine practice or how delivery influences patient engagement across different clinical settings.

**Objective:**

This study explored perceived barriers and facilitators to the delivery of and engagement with *myCOPD* by examining the perspectives of both patients and health care professionals (HCPs) across 2 National Health Service settings.

**Methods:**

This qualitative study was conducted between November 2023 and April 2024 across 2 contrasting clinical pathways: community pulmonary rehabilitation and a hospital discharge service following acute exacerbation of COPD. Semistructured interviews were conducted with patients using *myCOPD* and HCPs involved in its delivery. Topic guides covered health background, digital technology use, and experiences of engaging with the intervention. A convenience sampling approach, with elements of purposive sampling, ensured representation across settings. Clinical data were obtained from health records with consent, and usage data were provided by my mhealth Ltd. Data were analyzed using abductive thematic analysis informed by the Medical Research Council’s process evaluation framework.

**Results:**

Thirty interviews were completed (16 patients and 14 HCPs). Patients described how multimorbidity, perceived digital ability, and socioeconomic context shaped their engagement with *myCOPD*, operating as “layers of vulnerability” that influenced whether COPD self-management could be prioritized. HCPs highlighted organizational and workflow constraints that shaped how the intervention was introduced and supported. Delivery approaches differed markedly between settings: community teams adopted proactive, relationship-based support that enabled iterative discussion, tailored recommendations, and continuity, whereas hospital teams described passive, onboarding-focused delivery shaped by time pressures, staffing constraints, and discharge priorities. These contrasting delivery models influenced engagement patterns, with proactive support associated with more *multifaceted engagement* (characterized as routine and sustained) and passive onboarding associated with *single-aspect engagement* (characterized as short-term and transactional). Participants also identified perceived barriers (eg, usability concerns, limited integration with in-person care) and perceived benefits (eg, improved inhaler technique, increased confidence, lifestyle adjustments).

**Conclusions:**

Delivery context and local workflows play a central role in shaping how patients engage with digital self-management tools. Proactive, relational support appears to facilitate deeper and more sustained engagement, whereas passive onboarding may limit the intervention’s potential. Implementation strategies should align digital tools with local capacities, staffing structures, and patient characteristics, attending to layered vulnerabilities such as multimorbidity and digital confidence. Further work is needed to understand how proactive delivery models can be resourced and integrated within routine COPD care.

## Introduction

Chronic obstructive pulmonary disease (COPD) affects an estimated 3 million people in the United Kingdom and contributes to approximately 30,000 deaths annually, many of which are preventable [1,2]. Exacerbations are clinically significant events that increase the risk of subsequent exacerbations and hospital readmissions, particularly within the first 3 months [3,4]. Effective self-management, including symptom recognition, inhaler technique, and timely action, can reduce exacerbations and improve quality of life [5,6]. However, access to pulmonary rehabilitation (PR), a core component of COPD self-management, remains limited, with fewer than one-third of eligible patients accessing PR within 90 days of referral [7]. These persistent gaps highlight the need for scalable approaches to support self-management across care pathways.

Digital self-management interventions offer a potential means to extend PR and provide ongoing support, yet evidence for their effectiveness remains mixed [8-10]. While some studies report improvements in inhaler technique, symptom monitoring, and exercise adherence, others highlight challenges related to uptake, sustained engagement, and integration into routine care. A recurring issue is that digital interventions are often implemented without sufficient understanding of how they fit within existing clinical workflows or how frontline teams introduce and support their use [11]. Implementation research emphasizes that digital tools are not inherently effective; their impact depends on how they are delivered, supported, and embedded within organizational contexts. Factors such as staffing capacity, digital readiness, professional roles, and local service priorities can shape adoption, fidelity, and engagement [12].

Engagement with digital interventions is increasingly conceptualized as an implementation outcome influenced by both individual and structural determinants. COPD disproportionately affects older adults and people experiencing socioeconomic disadvantage, who may also face barriers related to digital access, confidence, and multimorbidity [13,14]. These overlapping factors can shape how individuals navigate digital tools and the extent to which they are able, or supported, to engage with them [15,16]. Understanding how patient characteristics intersect with service-level contexts is therefore essential for ensuring equitable benefit. An intersectional approach, attending to how multiple characteristics combine to produce advantage or disadvantage, can illuminate how patients navigate digital tools within the constraints of multimorbidity, digital ability, and socioeconomic context [11,17,18]. The concept of “layers of vulnerability” proposed by Luna [19,20] is particularly relevant for understanding how vulnerabilities emerge from the interaction between individuals and their health care environments, rather than from individual characteristics alone.

*myCOPD* is a widely adopted digital self-management intervention recommended by the NICE Early Value Assessments for PR and COPD self-management [21]. Despite its deployment across National Health Services (NHSs), limited evidence exists on how *myCOPD* is implemented in real-world settings, how delivery varies across clinical pathways, or whether these variations influence patient engagement. Existing evaluations have focused primarily on clinical outcomes or usability, with less attention to the organizational and relational processes that shape engagement in practice [22-25].

To address this gap, we examined the implementation of *myCOPD* across 2 contrasting NHS settings: community PR and a hospital discharge pathway following an acute exacerbation of COPD. Guided by the Medical Research Council’s (MRC’s) process evaluation framework [26], we explored how contextual factors, delivery approaches, and patient characteristics interact to shape engagement trajectories. This study aimed to identify implementation factors influencing the delivery of and engagement with *myCOPD* across 2 NHS clinical settings and explore how patient characteristics and structural factors interact to shape engagement trajectories, informed by the concept of “layers of vulnerability.”

By analyzing implementation processes across settings, this study contributes to our understanding of how digital self-management interventions can be more effectively and equitably integrated into routine COPD care. These aims informed the sampling strategy, interview topic guides, and abductive analytic approach.

## Methods

### Study Settings

The study was conducted across 2 contrasting NHS clinical settings:

First, the hospital discharge pathway (Bristol), where *myCOPD* was introduced as part of the acute exacerbation of COPD discharge bundle. Eligible patients were identified during follow-up clinics or virtual ward reviews within 6 weeks of hospital discharge. Delivery was primarily supported by digital health champions (DHCs) due to staffing pressures and workflow constraints.

Second, the community PR (Cornwall), where *myCOPD* was integrated into routine PR delivery. A digital health advisor (from my mhealth Ltd) and a respiratory care coordinator supported registration, activation, and ongoing use. Clinicians used the *myCOPD* dashboard to monitor progress and tailor self-management discussions.

These settings differed in purpose, workflow, and available resources, providing a natural comparison of delivery approaches.

### Intervention: *myCOPD*

*myCOPD* is a digital self-management app providing educational modules, inhaler-technique videos, PR content, symptom tracking, and action-plan support. Clinicians can view patient-reported data through a dashboard to support remote monitoring and personalized care. The intervention content was identical across sites.

### Participants, Recruitment, and Sampling

Patients were recruited from the PROPEL (A Pragmatic Real-World Multicenter Observational Research Study to Explore the Clinical and Health Economic Impact of *myCOPD*) study cohort. During their baseline PROPEL visit, all eligible participants were informed about the qualitative component and invited to opt in or out. Those who opted in were recontacted to arrange an interview after their scheduled 3-month follow-up visit, timed to ensure adequate exposure to the intervention. The eligibility criteria at each site are outlined in Textbox 1.

Textbox 1. **Eligibility criteria across settings.**

**Setting 1 (Bristol)**

- **Inclusion criteria**
  - Adult patients aged >18 years and able to provide informed consent
  - Clinical diagnosis of chronic obstructive pulmonary disease (COPD)
  - Admitted to hospital with a primary diagnosis of acute exacerbation of COPD (AECOPD)
  - Assessed in a follow-up clinic or a virtual ward within 6 weeks of an AECOPD

- **Exclusion criteria**
  - Patients aged <18 years
  - No clinical diagnosis of COPD
  - End-of-life care or palliative care
  - Unable to provide informed consent

**Setting 2 (Cornwall)**

- **Inclusion criteria**
  - Adult patients aged >18 years and able to provide informed consent
  - Clinical diagnosis of COPD, deemed suitable for referral to pulmonary rehabilitation by the local clinical team
  - Motivated or willing to take part

- **Exclusion criteria**
  - Unstable angina
  - Myocardial infarction within 6 weeks
  - Uncontrolled cardiac arrhythmias
  - Unstable hypertension
  - Severe cognitive impairment
  - Locomotor or other severe medical conditions preventing the patient from safe participation in group activities
  - Unable to provide informed consent
  - Any condition deemed by the principal investigator to make the participant unsuitable for the study

A convenience sampling approach was used, with elements of purposive sampling to ensure representation across both clinical settings. Health care professionals (HCPs) involved in delivering *myCOPD* were identified by site leads and approached via email. A purposive sampling strategy ensured variation in professional role (DHCs, nurses, and physiotherapists) and setting (community vs hospital).

### Data Collection

Semistructured interviews were conducted via Microsoft Teams (audio or video, depending on the technological accessibility of participants). Topic guides were tailored for patients and HCPs and refined iteratively by experienced qualitative researchers. Interview topics included experience of COPD, use of digital technology, engagement with *myCOPD*, and perceived barriers and facilitators. Example questions are presented in the full topic guides in Multimedia Appendix 1.

With informed consent, clinical data (eg, COPD severity, years since diagnosis, long-term conditions) were obtained from participants’ health records held by NHS clinical teams. Similarly, with consent, *myCOPD* usage data (eg, logins, module completion, and PR access) were obtained directly from my mhealth Ltd, which provided anonymized usage summaries for participants enrolled in the PROPEL study.

### Procedure

At the start of each interview, participants received a verbal recap of study aims, confidentiality, and their right to withdraw. One interviewer was present at each interview (MR conducted all patient interviews, and HCP interviews were split between BC and MR). MR (a PhD researcher in health psychology with training in qualitative interviewing and prior experience as a patient and public involvement [PPI] officer) conducted all patient interviews and facilitated PPI sessions. BC (an experienced qualitative researcher) conducted HCP interviews. Their positionalities—MR’s long-standing ties to the local community and BC’s research background—were acknowledged and reflexively considered throughout the analysis. Interviews were recorded, transcribed verbatim, checked for accuracy, and imported into NVivo 14 (Lumivero) for analysis. All participants received financial compensation (payments of £75 [US $100] for HCPs; vouchers of £25 [US $30] for patients). Finally, the data presented here include CAT scores that were taken at baseline by clinical research staff.

### Analysis

Coding was conducted by 2 researchers (BC and MR) for HCP interviews and by a researcher (MR) for patient interviews. HCP and patient transcripts were coded independently. During theme development, we compared the coded data to identify shared patterns, at which point the analytical process merged into a unified set of themes. Analyzing the transcripts together captured the relational aspects of self-management and reflected an overlap in coding and candidate themes across groups. While both groups discussed a particular theme, typically one group described the theme in more detail; for example, HCPs and patients both described the importance of multimorbidity, but patients described in greater detail how it shaped engagement.

Researcher positionalities were addressed through structured reflexive practices. BC and MR kept journals during data collection and coding, revisiting these reflections during theme development to check for assumptions and bias. MR also held regular debrief meetings with senior qualitative researchers (BA, KB, and LY) to discuss how background experiences might shape interpretation. These discussions helped mitigate potential limitations of single coding and promoted consistency and rigor. MR also used analytic memos and diagramming to track decision-making and consider alternative interpretations. Together, these practices provided a systematic reflexive process that helped ensure interpretations remained grounded in the data.

Coding followed an abductive thematic analysis [27] workflow that combined the MRC process evaluation framework [26] with inductive code generation. Transcripts were first read repeatedly to support familiarization. A deductive coding structure based on MRC domains (context, implementation, engagement, mechanisms of impact) [26] served as the overarching structure, and data were coded line-by-line according to the relevant categories. Alongside this, inductive codes were generated to capture participant-led insights that did not fit the MRC framework. The coding framework was refined iteratively, with codes merged, repositioned, or expanded as patterns became clearer. Coded data were then reviewed within and across domains to identify candidate themes, with the MRC structure providing the overarching scaffold and inductive codes supplying the detailed content. Themes were developed through team discussion, diagramming, and matrix mapping to examine relationships across settings and between patient and HCP accounts. Reporting of qualitative methods and findings was informed by the COREQ (Consolidated Criteria for Reporting Qualitative Research) checklist (Checklist 1). This iterative movement between empirical data and implementation theory supported the identification of mechanisms, such as how delivery approaches shape engagement trajectories, that are central to understanding implementation and align with calls for qualitative methods [28].

### PPI

PPI activities were integrated throughout the PROPEL study and used to refine qualitative findings. The PPI group for the work outlined here consisted of Windrush Generation Elders (pioneering individuals who immigrated to the United Kingdom from Caribbean countries and other Commonwealth nations between 1948 and 1971). They all had experience of living with or caring for someone with asthma or COPD. This group highlighted the importance of intersectionality before data collection and therefore supported this focus. One-to-one sessions were held with stakeholders (professionals based in the NHS and at the digital provider) who were experienced in delivering *myCOPD*. Work with the community-based group adopted a participatory approach to activities (facilitated by MR). These activities were designed to assess the relevance, clarity, and real-world applicability of emerging themes. Feedback informed the refinement and interpretation of themes.

The PPI group was not told the results at the beginning of the workshop (Multimedia Appendix 2 provides further detail using GRIPP2 [Guidance for Reporting Involvement of Patients and the Public, version 2] form); they were asked to individually draw journey maps of their experiences with COPD (in some cases, asthma). We then discussed their maps, with the chance for them to respond to each other and adapt their own maps. Finally, the results were introduced, and we discussed factors that overlapped with the results and those that were different, reasons for differences, and the importance of each factor. Consent was not obtained for PPI activities, and therefore these do not feature as a facet in the “Results” section. PPI highlighted that the results were relevant, how they related to lived experience, and a few aspects that were missing from the results (eg, the role of carers).

### Ethical Considerations

Ethical approval was granted by the NHS Research Ethics Committee (REC reference: 23/SC/0155) and the University of Southampton (ERGO: 84797). All participants provided informed consent. Clinical and usage data were anonymized or deidentified before analysis. Confidentiality was maintained throughout, and participants received financial compensation for their time.

The qualitative interview data generated and analyzed during this study are not publicly available due to confidentiality agreements and the absence of participant consent for data sharing.

## Results

### Descriptive Data

Thirty interviews were conducted, 16 with patients and 14 with HCPs. Patient interviews lasted 14 to 35 (mean 23.7, SD 6.3) minutes, and HCP interviews lasted 23 to 57 (mean 36.8, SD 7.9) minutes. Sixteen of the 37 patients who initially agreed completed interviews; there was a range of reasons for nonparticipation: no response to invitations (n=15), unavailable contact details (n=2), technical failures (n=2), or withdrawal (n=2). Patient characteristics are provided in Table 1, and HCP characteristics are provided in Table 2. Participant usage data (Table 3) indicated higher engagement among Cornwall patients, who had a longer period since activation (107‐148 days) and more frequent logins. Across both sites, 81.25% (13/16) accessed educational modules and 68.75% (11/16) accessed PR content.

**Table 1.** Patient sample characteristics.

Site	Number, n	Age (y), mean (SD)	COPD^a^ severity, n			Females, n	White patients, n	Years since diagnosis, mean (SD)	Long-term conditions, n				Long-term conditions, mean (SD)	Lives alone, n	Smokers, n
			Mild	Moderate	Severe				0	1	2	3+			
Bristol	8	68.38 (5.32)	3	2	3	3	8	8.63 (5.13)	1	1	2	4	2.38 (1.41)	3	2
Cornwall	8	66.50 (7.95)	5	3	0	3	8	8.38 (8.11)	1	2	1	4	3.25 (2.76)	1	3
Total	16	67.44 (6.60)	8	7	3	6	16	8.50 (6.55)	2	3	3	8	2.81 (2.17)	4	5

a COPD: chronic obstructive pulmonary disease.

**Table 2.** Summary of HCPs^a^ interviewed^b^.

Site	Number, n	White HCPs, n	Digital health champion, n	Nurse or physiotherapist, n
Bristol	8	8	3	5
Cornwall	6	5	1	5
Total	14	13	4	10

a HCPs: health care professionals.

b One digital health champion also worked as a senior physiotherapist (Cornwall), and another had previously worked as a nurse (Bristol).

**Table 3.** Summary of usage data.

Site	Number, n	CAT^a^ score at baseline, mean (SD)	Days since activation, mean (SD)	Number of login days, mean (SD)	Patients accessing education modules, n (%)	Education completion, mean (SD)	Patients accessing PR^b^ modules, n (%)	PR modules accessed, mean (SD)
Bristol	8	18.29 (6.78)	168.75 (88.06)	49 (69.10)	6 (75.0)	51.6 (44.5)	5 (62.5)	2.8 (1.66)
Cornwall	8	18.09 (8.12)	148.63 (48.29)	57.88 (41.26)	7 (87.5)	58.6 (41.0)	6 (75)	2.5 (1.72)
Total	16	18.19 (7.22)	158.69 (69.39)	53.44 (55.17)	13 (81.3)	52.4 (41.3)	11 (68.75)	2.65 (1.28)

a The COPD Assessment Test (CAT) is designed to measure the impact of chronic obstructive pulmonary disease (COPD) on a person’s life and assess changes over time: 0‐9: low impact; 10‐20: medium impact; 21‐30: high impact; and 31‐40: very high impact.

b PR: pulmonary rehabilitation.

### Overview of Thematic Analysis

Five themes were developed, each with subthemes (Table 4). Read sequentially, the themes describe a pathway from patient characteristics to delivery context, engagement patterns, perceived barriers, and perceived benefits. This structure highlights how individual and structural factors interact to shape engagement with *myCOPD*. As mentioned in the “Methods” section, the analysis of the transcripts was often complementary between HCPs and patients. For example, both HCPs and patients could describe the health care setting as affecting engagement with *myCOPD*, though HCPs typically provided more detail.

**Table 4.** Summary of themes and subthemes.

Theme	Subthemes	Interplay between HCP^a^ and patient accounts
Patient characteristics related to engagement	Perceived digital ability Negotiating multimorbidity Socioeconomic status	Complementary accounts, patients provided more detail
Important differences in health care setting	Perceived purpose of health service Organizational-structural factors Approaches to delivery of *myCOPD*	Complementary accounts, HCPs provided more detail
Engagement with *myCOPD*	Single-aspect engagement Multifaceted engagement	Complementary accounts, patients provided more detail
Perceived barriers to engagement	User perceptions of *myCOPD* External barriers	Complementary accounts Users provided more detail HCPs provided more detail
Perceived benefits of engagement	Targeted behavior change Wider lifestyle adjustments	Complementary accounts, patients provided more detail

a HCP: health care professional.

### Patient Characteristics Related to Engagement

Three characteristics (multimorbidity, perceived digital ability, and socioeconomic context) operated as “layers of vulnerability” influencing engagement. Importantly, it was not the presence of a characteristic alone but how patients interpreted and negotiated that characteristic that shaped their engagement trajectory.

#### Negotiating Multimorbidity

The impact of living with multiple long-term conditions was commonly discussed. HCPs and patients often recognized the uncertainty caused by negotiating multimorbidity.

*It’s very difficult, when you’ve got another illness, you can’t really put it all on one thing.*

[PAT06, hospital patient]

Other health conditions potentially inhibit engagement with *myCOPD*. For example, positive management of another condition could affect quality of life to such an extent that COPD was not currently prioritized (eg, weight loss). In other instances, the presentation of other health conditions seemed to compete for prioritization.

*I’ve got asthma, COPD, the heart problem, and I’m being treated for bladder cancer. Unfortunately, it’s [COPD is] like number three [in terms of priorities].*

[PAT03, hospital patient]

These accounts illustrate how multimorbidity among patients formed a core layer of vulnerability, shaping whether COPD self-management could be prioritized and how patients navigated competing health demands.

#### Perceived Digital Ability

Patients’ perceptions of their own digital ability also impacted engagement with *myCOPD*. Some participants expressed a level of digital inhibition that prevented engagement. Patients with conditions such as dyslexia could also perceive engagement as overwhelming.

*I’m afraid of it...I don’t know what I’m doing half the time. I’ll get confused. I don’t want to put the wrong answer...it worries me.*

[PAT05, community patient]

A common contradiction was observed among patients between their perceived digital abilities and their reported digital capabilities. The following quote is from a patient who described their use of digital technology as “not good” and “very limited.”

*[I have] an iPhone 13 mini...I go on Internet, social media occasionally...check football scores...I use Google quite a lot. I’ll check the weather and watch videos...Occasionally I’ll check the news. [And I use] WhatsApp...I use the NHS app to order prescriptions...I find that easier to do [with] the NHS app, probably easier than trying to get to see someone.*

[PAT08, hospital patient]

Perceived digital ability operated as another layer of vulnerability, influencing not only whether patients felt able to use *myCOPD* but also how confidently they interpreted and acted on its content.

#### Socioeconomic Status

Socioeconomic status (SES) was rarely discussed directly, and a marker of SES was not collected as part of the PROPEL study, but there were instances where economic resources appeared to influence engagement with *myCOPD*. The following extract highlights a participant of high SES who was less invested in the app because they were able to access support for self-management from a private health care team.

*I couldn’t keep waiting for NHS responses, so I went privately to see a respiratory consultant, and he put me onto an ENT consultant who is very good. And they also put me onto a respiratory physio, and she was very good. And I’ve obviously taken hints from them of what to do. And yeah, I think I’ve got most things under control fairly well.*

[PAT09, community patient]

Socioeconomic context, specifically having the financial ability to seek private health services, presents a potential layer of vulnerability. Such differences in financial resources may influence the perceived value of engaging with *myCOPD*.

### Important Differences in the Health Care Setting

#### Approaches to Delivering *myCOPD*

This theme highlights factors relevant to how HCPs approached the delivery of *myCOPD*. Two subthemes (the perceived purpose of the health service and organizational-structural factors) appeared to influence the third subtheme (approaches to the delivery of *myCOPD*). Given the distinct approaches to delivering *myCOPD*, the health care setting is conceptualized as a layer of vulnerability that can hinder or promote engagement with *myCOPD*. These approaches also influenced how clinical teams used DHCs.

#### Community Setting

Among community professionals, the general perception was that the purpose of their service was to provide and improve COPD self-management regimens over several weeks. Community professionals often described discussing relevant self-management strategies with patients and using *myCOPD* as an initial step toward engagement.

*[We] try and find out which is the best route initially [for the patient to] start engaging with the app...I suggest looking at chest clearance, or if they’re not taking inhalers properly then we’d look at the inhaler technique...trying to find something that they engage with well and get them to concentrate on that initially.*

[HCP12, community-based HCP]

Between contacts, community professionals described checking patient usage reports in *myCOPD*. At in-person visits, professionals would check patient responses to, and understanding of, accessed content and then develop self-management strategies by signposting patients to further relevant content. In this context, DHCs were used for brief check-ins with patients and to address technical issues. This proactive approach was often described as a clear and purposeful process that used *myCOPD* as a medium to demonstrate and discuss tailored self-management techniques.

*I do like [myCOPD] because you can see how well the patients are doing with that symptom tracker...you can have a look at your patients and say “oh, he’s not good today. It might be worth giving him a phone call,” [to ask the patient] “have you spoken to a GP, have you taken action on this?”*

[HCP11, community-based HCP]

#### Hospital Setting

In contrast, hospital professionals often described a service that focused on recovery from exacerbation in preparation for discharge. Organizational-structural factors appeared to influence the approach to delivering *myCOPD*. For example, hospital-based professionals perceived internet connectivity, understaffing, and patient expectations (of being discharged) as adversely affecting how *myCOPD* was delivered.

*There’s quite a big workload for my colleagues, [it’s] quite a big hospital. If there was maybe more employees to go through the app...but I don’t know if we’ve got enough resources...to thoroughly implement it.*

[HCP7, hospital-based HCP]

These contextual issues induced an approach to delivery that was passive, fundamentally reducing engagement to onboarding. This was commonly expressed as “selling” *myCOPD*. This passive approach seemed to be an embedded process in which clinical professionals avoided discussing the app with patients; anything related to the app was deemed the work of the DHC.

*We ask the Band 4 [DHC] to see the patients for the myCOPD app, you know introduce [it and] phone them again afterwards.*

[HCP1, hospital-based HCP]

### Engagement With *myCOPD*

This theme describes how patients perceived engagement with *myCOPD*, a perception seemingly affected by contextual factors described in previous themes. Two subthemes are used to describe different types of engagement. The contrast in engagement across the 2 sites highlights that engagement itself is a layer of vulnerability rather than a neutral process, one that does not rely solely on design and individual capacity.

#### Single-Aspect Engagement

Single-aspect engagement describes a focused type of engagement, characterized by accessing specific parts of the app, as if they were stand-alone components (eg, breathing exercises, inhaler technique, and physical activity). Single-aspect engagement was often described in terms that were transactional and short-term, patients expected to achieve clear benefits from use. Both sets of patients, community and hospital, described single-aspect engagement.

*[Regarding inhaler technique] I didn’t realize I was doing so much wrong, and it does make a difference if you do it properly...[also] some of the techniques for getting your breath back [I] didn’t know anything about that. Again, that’s something new that I’ve learnt.*

[PAT06, hospital patient]

#### Multifaceted Engagement

Multifaceted engagement describes a more comprehensive use of *myCOPD*, often characterized by a sense of purpose and routine. Single-aspect engagement remains present, but usage does not need to fulfill a specific function.

*It’s a normal daily thing. You know, I do it in the morning to record when I take my medicines and steps, and I do it in the evening to record any exercise I’ve done.*

[PAT04, community patient]

Multifaceted engagement seemed to stem from proactive communication and management between patients and professionals. Hospital HCPs tended to check and prompt usage, whereas community HCPs tended to check and prompt meaningful engagement. Here, interviews with HCPs help to understand the different types of engagement described by patients.

*I see if they’re actually using it [myCOPD], and if not, I do tend to send them a little message...You know, “Please remember to use this” or “I have noticed you haven’t activated it” or “I’ve noticed you haven’t used it in a couple of weeks.”*

[HCP13, hospital HCP]

*I’ll use the app to check how the patients doing and then motivate them on these follow up calls, to motivate them how to continuously use [myCOPD] because the whole point is to make sure they’re getting that PR support after an exacerbation. So, to ensure they don’t get unwell and really focus.*

[HCP2, community-based HCP]

### Perceived Barriers to Engagement

#### Overview

Perceived barriers to engagement seem to be affected by the previous themes and are categorized into 2 subthemes. User perceptions of *myCOPD* describe gaps between the actual provision of *myCOPD* and users’ understanding of that provision. External barriers are perceived issues that are beyond the direct remit of *myCOPD* (eg, fit with in-person care and the degree to which *myCOPD* is embedded within services). Misunderstandings about app functionality and uncertainty about data fidelity interact with existing layers of vulnerability, shaping how confidently patients and clinicians rely on the tool.

#### User Perceptions of *myCOPD*: App Functionality

While many participants (both patients and HCPs) described *myCOPD* as easy to use, some participants made initial judgments based on whether they perceived the app as intuitive. For example, some participants passed judgment based on their understanding of a specific function being absent (eg, accessibility functions) and not realizing that the desired functionality was offered.

*Patients who cannot read, [there is a] low reading age in Cornwall, or [have] eyesight problems, or have dyslexia may struggle as the questionnaires and tabs are all word-based. Perhaps a speaker icon with the questions and tabs narrated would benefit patients.*

[HCP11, community HCP]

This perceived functionality was described by some participants when navigating *myCOPD*. These perceptions could have been overcome through discussion with technical support. Similarly, some patients described burdensome expectations to complete “lengthy” physical exercise sessions, even though *myCOPD* typically builds tolerance, ranging from 8 to 40 minutes, depending on user preferences.

*I’ve just been through the exercises and it’s an hour’s worth of exercises...the whole bundle of exercises and watching all the videos and doing the exercise is close on an hour.*

[PAT09, community patient]

#### User Perceptions of *myCOPD*: Trust in Patient Usage Reports

Community-based patients and professionals articulated the importance of building trust in patient self-reports provided in *myCOPD*. This trust would encourage professionals to tailor treatment effectively. Simply providing reports without an explanation of their underlying fidelity left room for suspicion. For example, the Activity Diary in *myCOPD* has a calendar option that reports the number of minutes and the Rating of Perceived Exertion achieved during exercise, a design aimed at increasing fidelity.

*[Usage reports] tell you that patients have done their PR, but actually they opened the tile, looked at the first page and then ticked that they’ve done all the exercise. You get that clinician’s sixth sense, that they might not have actually done the exercise.*

[HCP9, community-based HCP]

#### External Barriers: Contrasting Perceptions of Fit With In-Person Care

Patients did not expect HCPs to check their *myCOPD* activity but also felt unable to increase their engagement in conversations during face-to-face consultations. As outlined above, community professionals frequently described using *myCOPD* to better target their professional expertise. In contrast, hospital professionals often preferred in-person care to using *myCOPD*.

*We see them after the acute phase, they’ve had an exacerbation, and they’re in hospital and I suppose generally that’s always gonna be better...It’s better to see anyone face-to-face.*

[HCP7, hospital-based HCP]

#### External Barriers: Embedding *myCOPD* Into Routine Practice

Professionals frequently mentioned that *myCOPD* was not embedded enough within services. Embedding sometimes meant having a critical mass of users to be seen as routine practice; at other times, it meant buy-in from frontline staff and managers.

*I think it needs to be enforced by the whole team whereby we say [to patients], “look, when you’re going home, we need to see this information and it’s gonna be used in the clinic.” I think then...adherence would be better.*

[HCP6, hospital-based HCP]

Community HCPs were proactively using *myCOPD* but also highlighted the need to provide a dedicated space to support patients with engagement during existing face-to-face consultations.

*If it [myCOPD] was part of pulmonary rehab and we did make it more approachable, [it would] make them [patients] feel like they’re all doing it together, they’re all in the same boat [as COPD patients]...a bit of support over the next few weeks, then you’ll be good to run with it.*

[HCP3, community-based HCP]

Where *myCOPD* was not well integrated into routine care, organizational constraints compounded existing layers of vulnerability, limiting opportunities for patients to develop sustained engagement.

### Perceived Benefits of Engagement

The perceived benefits of engagement with *myCOPD* appear to be affected by the first 3 themes. The first subtheme captures direct benefits associated with *myCOPD*. For HCPs, this meant positive alignment with existing care guidelines; for patients, this meant that the app was a tangible resource for improving specific abilities. The second subtheme describes wider benefits of *myCOPD*.

#### Targeted Behavior Change

Targeted behavior change describes how *single-aspect engagement* was perceived to improve specific abilities (eg, breathing through an exacerbation, improved inhaler technique, and increased physical activity). This association often led to descriptions of improved self-confidence. Overall, patients described being able to use *myCOPD* to address and improve specific behaviors that concerned them.

*It’s [chest clearance on myCOPD] helped...that’s one thing, you can hear it now. I’ll get like a frog in the throat and in the chest...everybody thinks I’ve got cough or cold all the time, but hang on, just got to clean myself [performs chest clearance].*

[PAT03, hospital patient]

*[I’ve got] more confidence, really, confidence in managing my condition...When I used to walk uphill, to do the horses, [I] used to stop to take a breath or take my inhaler and now I just keep going but I slow down...I realise that I don’t have to stop, and I don’t have to rush...[I’m] kind of not worried about losing my breath because I can control it.*

[PAT07, community patient]

#### Wider Lifestyle Adjustments

This subtheme describes benefits associated with *multifaceted engagement*. This seemed to be a longer-term type of engagement with benefits yet to materialize. This type of benefit was only associated with patients from community settings and was typically developed through a latent process of analysis. Short transcript extracts do not adequately explain this subtheme; however, there was one example that demonstrated the potential benefits of wider lifestyle adjustments.

One patient noticed increasing breathlessness over several weeks and did not understand why. The patient eventually accessed the reporting functions in *myCOPD* and, because they had been recording exercises and symptoms daily, were able to spot a pattern in their breathlessness. Consequently, they were able to deduce that the breathlessness was a result of chopping wood and adjusted their behavior to avoid symptoms by taking a preventative inhaler before chopping wood or delaying the activity until later in the day.

## Discussion

### Principal Findings

This study explored how *myCOPD* was delivered and used across 2 contrasting NHS settings and how contextual, organizational, and individual factors shaped engagement. Across both sites, patients described how multimorbidity, perceived digital ability, and socioeconomic context influenced their capacity to engage with the intervention. These characteristics did not operate in isolation; rather, they interacted with local delivery practices to shape engagement trajectories.

A key finding was the marked difference in delivery approaches between settings. Community PR HCPs adopted a proactive, iterative model of support using *myCOPD* to structure conversations, reinforce self-management strategies, and monitor progress. In contrast, hospital HCPs described a more passive, onboarding-focused approach shaped by workflow pressures, limited staffing, and the prioritization of discharge. These delivery styles appeared to influence whether patients engaged in single-aspect engagement (targeted, short-term usage) or multifaceted engagement (routine usage).

Both HCPs and patients identified barriers and facilitators to engagement. Barriers included usability concerns, competing health priorities, limited digital confidence, and challenges integrating the app within in-person care. Facilitators included personalized support from clinicians, clear explanations of app features, and opportunities to discuss content during follow-up contacts. PPI contributors confirmed the relevance of these findings, emphasizing the importance of timely support, clear communication about the purpose of the app, and recognition of the challenges posed by multimorbidity and limited digital confidence.

A key strength of this study is the inclusion of both patients and HCPs across 2 contrasting NHS pathways, enabling a nuanced understanding of how delivery context shapes engagement. The use of abductive thematic analysis allowed us to integrate theoretical insights with inductively generated themes, and PPI involvement strengthened the relevance and clarity of the findings. Collectively, these findings highlight that engagement with *myCOPD* is not simply a function of individual motivation or app design but emerges from the interaction between patient characteristics and the delivery environment, an insight that aligns with and extends implementation theories emphasizing context-mechanism interactions [27].

### Comparison With Prior Work

Our findings align with implementation research demonstrating that digital interventions are not inherently effective; their impact depends on how they are introduced, supported, and embedded within clinical workflows [11,26]. Prior COPD digital evaluations [22-25] similarly reported high initial interest in inhaler videos and symptom tracking but noted rapid declines in use. Our findings extend this work by showing why this decline occurs, specifically, that passive onboarding in hospital settings limits opportunities for relational reinforcement.

More specifically, our observation that proactive, relationship-based support in community PR settings facilitated sustained engagement echoes the work of Pinnock et al [5,6] on supported self-management and aligns with the theorization of implementation mechanisms by Lewis et al [28], which emphasizes the importance of relational reinforcement and iterative feedback loops. Our data provide concrete examples of how these mechanisms operate within COPD pathways, illustrating how clinicians used *myCOPD* to scaffold conversations, tailor recommendations, and maintain continuity, practices that have been theorized but rarely described empirically in COPD digital implementation studies.

Conversely, the passive onboarding approach observed in hospital settings reflects challenges documented in acute care environments, where digital tools are often introduced during periods of high patient burden and limited staff capacity [10,14]. This is consistent with the findings of O’Connor et al [29], which indicate that workflow pressures and resource constraints in acute care limit opportunities for relational support and follow-up. Our findings extend this literature by showing how these constraints shape not only uptake but also the depth and trajectory of digital engagement, with hospital patients more likely to adopt single-aspect, short-term use.

Our findings also contribute to emerging evidence on digital inequalities. Multimorbidity, perceived digital ability, and socioeconomic context shaped how participants navigated *myCOPD*, consistent with the literature highlighting the intersection of health and digital disparities [13-16]. Informed by the concept of “layers of vulnerability” proposed by Luna [19,20], our analysis suggests that these vulnerabilities are dynamic and relational, shaped not only by individual circumstances but also by the support available within clinical settings. This was evident in patients who described shifting their self-management priorities in response to exacerbations or competing long-term conditions, relying on family members or clinicians to navigate digital tasks when confidence fluctuated, and adjusting their engagement depending on whether services provided opportunities for discussion, troubleshooting, or reinforcement.

In doing so, our study provides empirical grounding for the conceptualization of vulnerability as layered rather than categorical, as proposed by Luna [19,20], demonstrating how multimorbidity, digital confidence, and socioeconomic constraints accumulate and interact within real-world COPD pathways. This complements the call for intersectional approaches to digital health disparity research made by Husain et al [11] and aligns with the argument of Veinot et al [30] that digital inequalities are structurally produced rather than individually determined. Our findings also resonate with the findings of Greenhalgh et al [31], specifically the finding that multimorbidity often complicates digital adoption, particularly when tools are not embedded within supportive relational contexts.

### Limitations

Although this study offers important insights into how delivery context shapes engagement with *myCOPD*, several limitations should be acknowledged when interpreting the findings. First, participants were recruited from within the PROPEL study cohort, which may limit transferability to settings where *myCOPD* is introduced outside of a research context. Second, interviews were conducted remotely, which may have influenced participation among individuals with limited digital access. Third, while we examined differences across 2 settings, the sample size did not allow for detailed subgroup analysis (eg, by digital literacy, severity, or SES). Finally, usage data provided by my mhealth Ltd offered helpful context but did not capture qualitative nuances of engagement. These limitations underscore the need for future work that examines delivery models across a wider range of organizational contexts and includes more granular measures of digital literacy and multimorbidity burden, as recommended by Venoit et al [30].

### Implications for Practice and Implementation

Our findings highlight the importance of aligning digital self-management interventions with local workflows and capacities. Proactive, iterative support (such as checking usage data, discussing content during follow-up, and tailoring recommendations) appears to facilitate more meaningful engagement. Services adopting *myCOPD* may benefit from clarifying roles (eg, between clinicians and DHCs), ensuring adequate staffing, and embedding digital support within routine contacts. These implications arise directly from the contrast observed in our study: PR clinicians were able to provide iterative support because roles were clear and time was protected, whereas hospital clinicians lacked these structural conditions. More specifically, our data suggest that proactive delivery models may require explicit resourcing, including protected time for clinicians to review usage data and integrate digital discussions into routine care. This aligns with the argument of Greenhalgh et al [32] that digital health implementation depends on relational work and organizational readiness. Hospital settings, where workflow pressures limit such opportunities, may benefit from dedicated DHCs or structured onboarding protocols that extend beyond discharge.

At the patient level, recognizing the influence of multimorbidity, digital confidence, and socioeconomic context is essential for equitable implementation. Tailored onboarding, opportunities for hands-on support, and clear communication about the purpose and benefits of the app may help address barriers. These findings reinforce the value of attending to layers of vulnerability when designing and delivering digital self-management support. In practice, this may involve prioritizing hands-on demonstrations for patients with low digital confidence, offering multimorbidity-sensitive guidance that helps patients identify which app features align with their immediate priorities and ensuring that digital support is available beyond initial onboarding. These approaches are consistent with the suggestions of Vorrink et al [33] to improve digital self-management interventions.

For policy and commissioning, these findings suggest that digital interventions should not be implemented as stand-alone tools but as components of broader self-management pathways that account for organizational readiness and patient diversity. Commissioners may therefore need to incorporate workflow assessments, staffing considerations, and digital literacy support into procurement and implementation plans, ensuring that digital tools are embedded within coherent, adequately resourced pathways rather than added as isolated components. This aligns with Normalization Process Theory as outlined by Murray et al [34], which emphasizes the importance of integration within existing workflows.

### Implications for Research

Future research should examine how different models of delivery influence long-term engagement and clinical outcomes, including whether proactive support can be feasibly scaled. Further work is also needed to explore how digital interventions can be tailored for individuals with multimorbidity, low digital confidence, or limited access to technology. Mixed methods evaluations that integrate qualitative insights with detailed usage analytics may help identify mechanisms of engagement and inform more equitable implementation strategies.

Our findings point to several specific avenues for investigation, including comparative studies of proactive versus passive delivery models, evaluations of DHC roles within COPD pathways, and intersectional analyses that examine how layers of vulnerability shape engagement trajectories over time. Integrating relational measures of support with usage analytics may be particularly valuable for identifying mechanisms that drive sustained engagement, as suggested by the work of Lewis et al [28] on implementation mechanisms.

### Conclusions

Delivery context and local workflows play a central role in shaping how patients engage with *myCOPD*. Proactive, relationship-based support appears to facilitate more integrated and sustained engagement, whereas passive onboarding may limit the intervention’s potential. Considering patient characteristics, particularly multimorbidity and perceived digital ability, alongside organizational capacities may support more equitable and effective implementation of digital self-management tools in COPD care. The concept of layers of vulnerability provides a useful lens for understanding how individual and structural factors combine to shape engagement trajectories.

By illustrating how delivery models interact with patient characteristics to shape engagement, this study contributes to a more nuanced understanding of digital self-management implementation and highlights the need for context-sensitive, equity-oriented approaches to digital health in COPD care.

## Supplementary material

10.2196/88108 Multimedia Appendix 1 Topic guide.

10.2196/88108 Multimedia Appendix 2 GRIPP2 form.

10.2196/88108 Checklist 1 COREQ checklist.

## Abbreviations

COPD: chronic obstructive pulmonary disease
COREQ: Consolidated Criteria for Reporting Qualitative Research
DHC: digital health champion
GRIPP2: Guidance for Reporting Involvement of Patients and the Public, version 2
HCP: health care professional
MRC: Medical Research Council
NHS: National Health Service
PPI: patient and public involvement
PR: pulmonary rehabilitation
PROPEL: A Pragmatic Real-World Multicenter Observational Research Study to Explore the Clinical and Health Economic Impact of *myCOPD*
SES: socioeconomic status
