# Supplementary material for: Understanding Delivery and Engagement With a Digital Self-Management Intervention for Chronic Obstructive Pulmonary Disease Across Two Clinical Settings: Qualitative Study
Source: JMIR Hum Factors. 2026 Sep 21;13:e88108. doi: 10.2196/88108 (PMC13592746; doi:10.2196/88108)
Supplement: Supplementary file 2 [file humanfactors-v13-e88108-s001.docx]

**Topic Guide:** **Interviews - Experience of using myCOPD**

**Introduction and Confirmation of Consent**

| 1 | My name is… and I am…First of all, I would like to thank you for taking the time to talk to me today. As you know, we are interested in hearing about your experiences of the MyCOPD app. | ꙱ |
| --- | --- | --- |
| 2 | With your permission I will record the interview so that I am able to transcribe all that was said. If you want to stop the interview or recording at any time, you are free to do so. | ꙱ |
| 3 | Once the interview is transcribed, all names and identifying information will be removed to ensure anonymity. | ꙱ |
| 4 | You will be sent a copy of the transcript in case you wish to make clarifications or expand further on anything. | ꙱ |
| 5 | You can withdraw at any point during the interview. Just let me know. | ꙱ |
| 6 | Also, if it is okay with you, I would like to take some brief notes during the interview. | ꙱ |
| 7 | Are you happy to consent to take part in the study and for the interview to be recorded? | ꙱ |
| 8 | Before we begin do you have any final questions about the study? | ꙱ |
| 9 | Before we start, I want to stress that there are no right or wrong answers – I want to hear about your experiences. | ꙱ |

Example topics to cover in interviews:

- Perceptions of technology (for self-management).
- Experiences of MyCOPD.
- Using MyCOPD for pulmonary rehabilitation.
- Any interaction with digital health champions/health care professionals.
- Barriers and benefits to using MyCOPD.
- Any perceived benefits/impact of MyCOPD on self-management or symptoms.
- Opportunity to raise anything that they think we might have missed.

Questions Examples for Healthcare Professionals

Evaluating the implementation of myCOPD;

1. Please could you describe the typical process of using myCOPD with a patient, from the first session to the last?
2. Is this process very dfifferent to seeing patients in person?
3. Does the app fit well with existing care practices for COPD?
4. Have you experienced any problems when in using the app?
   a. Examples. Technological, communication, delivery of care.
5. Could you highlight any benefits of using the app?

Question Examples for Patient-Participants

1. Have you previously used technology to manage any aspect of your health?
   - Were you encouraged by using myCOPD?
2. How do you typically manage your COPD before using the app?
3. What is your experience of self-managing COPD?
4. What are your thoughts about using technology for self-managing your COPD?
5. Overall, what did you think of using myCOPD?
   - Were there any benefits?
   - Did you encounter any challenges?

Exploring Barriers to Using myCOPD (HCPs and patients)

Example questions:

1. What sort of challenges do you experience using MyCOPD?
2. Can you tell me about any practical barriers to using MyCOPD?
3. How do you feel about including MyCOPD in your daily routine (patients) /clinical practice (HCPs)?
4. Are there any ways that you think it could be made easier for you to use MyCOPD?

Exploring Benefits or Impact of myCOPD (HCPs and patients)

Example questions

1. Can you tell me about any impact that you think MyCOPD has had for you?
2. What do you think the benefits of using MyCOPD are?
3. Do you think that MyCOPD has changed the ways you self-manage (patients)/support COPD patients (HCPs) at all?

Future use of myCOPD (HCPs and patients)

Example questions:

1. How do you feel about using MyCOPD in the future?
2. What sort of things could be done to support your use of MyCOPD in the future?
3. Are there any barriers to your use of MyCOPD in the future?

Support of Digital Health Champions (HCPs and patients)

Example questions:

1. What is your experience of being supported by digital health champions?
2. How useful do you find the input of a digital health champion?
3. Can you tell me about whether you think the input of a digital health champion has changed your use of MyCOPD at all, and in what ways?

Implementation of myCOPD (HCPs and patients)

Example questions:

1. How did you find the rollout of MyCOPD?
2. Were there any barriers to the implementation of MyCOPD?
3. What did you think worked well about the implementation of MyCOPD?
4. What barriers do you think there will be for the use of MyCOPD long term?
5. Please can you tell me what you think about the implementation of MyCOPD in this particular area (e.g., Cornwall)? Any challenges specific to this location?
6. Do you feel able to use MyCOPD with the skills you already have?
7. How could the implementation of MyCOPD be improved in the future?
8. How could healthcare professionals be better supported in implementing MyCOPD?
