# Supplementary material for: Understanding Delivery and Engagement With a Digital Self-Management Intervention for Chronic Obstructive Pulmonary Disease Across Two Clinical Settings: Qualitative Study
Source: JMIR Hum Factors. 2026 Sep 21;13:e88108. doi: 10.2196/88108 (PMC13592746; doi:10.2196/88108)
Supplement: Supplementary file 3 [file humanfactors-v13-e88108-s002.docx]

[Gripp2 – Short Form](https://www.bmj.com/content/358/bmj.j3453)

PPI Session: PROPEL Study – Qualitative Aim (13^th^ January 2025)

***1: Aim***

*Report the aim of PPI in the study.*

The aim was to introduce myself, the study, and to facilitate activities that would stimulate discussion related to the study results. Crucially without presenting the results.

This was the first PPI session regarding the results of PROPEL.

The group has chosen to be referenced as, “The Priory Road Group”.

The session was held at a time convenient to the group at a location in central Southampton.

The group was made up of 14 Afro-Caribbean Elders (12 female, 2 male), all had asthma or COPD or experience of caring for or working in people with asthma or COPD.

**2: Methods**

*Provide a clear description of the methods used for PPI in the study.*

*Participatory journey mapping* is a creative and visual method used to allow participants to share their experiences of life with COPD.

Activity:

- Participants were given a live example of a journey map by the facilitator (MR). This example was unrelated to respiratory health.
- Participants were then asked to draw their own journey map focusing on their asthma history.
- Participants were not directed what to include and could discuss with each other.
- After 10 minutes participants were encouraged to look at each other’s maps and then consider if they wanted to further editing their own.
- After twenty minutes participants shared their journey map with the group.

The themes of the journey maps were relevant to the theme generated in study’s thematic analysis, any themes that was different or missing were discussed with the group.

**3: Study Results**

*Outcomes—Report the outcomes of PPI in the study, including both positive and negative outcomes.*

This PPI session added confidence in the results and highlighted limitations of the study. The session informed and shaped the discussion section but nothing specific was used.

**4: Discussion and conclusions**

*Comment critically on the study, reflecting on the things that went well and those that did not, so others can learn from this experience.*

The PPI session has encouraged the study to draw attention to the role played by carers and the need to ensure a diverse sample (not leaving this to chance but by design). This was a positive impact.

**5: Reflections/critical perspective**

Comment critically on the study, reflecting on the things that went well and those that did not, so others can learn from this experience.

The use of participatory journey mapping was positive, that added a sense of rigor to the results.

Generally, participants engaged well with the session and learned new things about each other, even though many have known each other for decades.
